# Engineering of *N. benthamiana *L. plants for production of N-acetylgalactosamine-glycosylated proteins - towards development of a plant-based platform for production of protein therapeutics with mucin type O-glycosylation

**DOI:** 10.1186/1472-6750-10-62

**Published:** 2010-08-24

**Authors:** Sasha M Daskalova, Josiah E Radder, Zbigniew A Cichacz, Sam H Olsen, George Tsaprailis, Hugh Mason, Linda C Lopez

**Affiliations:** 1 Center for Infectious Diseases and Vaccinology, The Biodesign Institute, Arizona State University, Tempe, AZ 85287, USA; 2 Center for Innovations in Medicine, The Biodesign Institute, Arizona State University, Tempe, AZ 85287, USA; 3 Center for Toxicology, University of Arizona, Tucson, AZ 85721, USA

## Abstract

**Background:**

Mucin type O-glycosylation is one of the most common types of post-translational modifications that impacts stability and biological functions of many mammalian proteins. A large family of UDP-GalNAc polypeptide:N-acetyl-α-galactosaminyltransferases (GalNAc-Ts) catalyzes the first step of mucin type O-glycosylation by transferring GalNAc to serine and/or threonine residues of acceptor polypeptides. Plants do not have the enzyme machinery to perform this process, thus restricting their use as bioreactors for production of recombinant therapeutic proteins.

**Results:**

The present study demonstrates that an isoform of the human GalNAc-Ts family, GalNAc-T2, retains its localization and functionality upon expression in *N. benthamiana *L. plants. The recombinant enzyme resides in the Golgi as evidenced by the fluorescence distribution pattern of the GalNAc-T2:GFP fusion and alteration of the fluorescence signature upon treatment with Brefeldin A. A GalNAc-T2-specific acceptor peptide, the 113-136 aa fragment of chorionic gonadotropin β-subunit, is glycosylated *in vitro *by the plant-produced enzyme at the "native" GalNAc attachment sites, Ser-121 and Ser-127. Ectopic expression of GalNAc-T2 is sufficient to "arm" tobacco cells with the ability to perform GalNAc-glycosylation, as evidenced by the attachment of GalNAc to Thr-119 of the endogenous enzyme endochitinase. However, glycosylation of highly expressed recombinant glycoproteins, like magnICON-expressed *E. coli *enterotoxin B subunit:*H. sapiens *mucin 1 tandem repeat-derived peptide fusion protein (LTBMUC1), is limited by the low endogenous UDP-GalNAc substrate pool and the insufficient translocation of UDP-GalNAc to the Golgi lumen. Further genetic engineering of the GalNAc-T2 plants by co-expressing *Y. enterocolitica *UDP-GlcNAc 4-epimerase gene and *C. elegans *UDP-GlcNAc/UDP-GalNAc transporter gene overcomes these limitations as indicated by the expression of the model LTBMUC1 protein exclusively as a glycoform.

**Conclusion:**

Plant bioreactors can be engineered that are capable of producing Tn antigen-containing recombinant therapeutics.

## Background

Plants emerged as effective systems for production of pharmaceutically important mammalian proteins, and a number of transgenic plant-based products are currently available in the market [1]. Work in the field of plant molecular farming revealed that, while there are obvious advantages to using plant bioreactors, including low cost, fast scalability, biological safety, proper protein folding and assembly, there are also some limitations. Glycosylation differences between plants and humans is one of the major drawbacks that can negatively influence properties of plant-derived recombinant proteins [2,3]. Significant efforts have been made to "humanize" plant N-glycan structures, either by retaining the recombinant proteins in the endoplasmic reticulum (ER) [4] or by modifying the enzyme machinery in the Golgi apparatus to eliminate plant-specific β(1, 2)-xylose and α(1,3)-fucose residues [5] and extend plant N-glycans with terminal β(1, 4)-galactose residues [6]. However, little attention has been paid to the structural differences between plant and mammalian O-glycans.

O-glycans are attached primarily to hydroxyproline (Hyp) and serine residues in plant glycoproteins. Typically, non-contiguous Hyp residues are modified with arabinogalactan polysaccharides, whereas contiguous Hyp are exclusively arabinosylated [7,8]. The extent of proline hydroxylation and Hyp glycosylation depends on the side chains of flanking amino acids [8]. A specific motif ([not basic]-[not T]-[AVSG]-Pro-[AVST]-[GAVPSTC]-[APS]) has been defined, based on examination of amino acid sequence requirements, for efficient hydroxylation of proline and arabinogalactosylation of Hyp [9]. No rules for selection of serine residues for O-glycosylation have been defined so far, probably because the number of identified proteins with glycosylated serines is still limited to a few plant lectins. Usually, plant O-glycans attached to serine residues are short, composed of galactopyranosides present either as monosaccharides or as 1→3-linked disaccharides [10,11]. In general, information about plant O-glycosylation enzyme machinery is lacking.

In contrast to plants, mammalian O-glycosylation is a well understood posttranslational modification. Substitutions of serine and threonine residues with sugars such as fucose, galactose and N-acetylglucosamine are common modification on mammalian proteins [12], but the most abundant O-glycans in mammalian cells are mucin type O-linked glycans. They are represented by structurally diverse oligosaccharides attached through N-acetylgalactosamine (GalNAc) to serine and/or threonine residues. Besides the initial GalNAcα-Ser/Thr, known as Tn antigen, other structures, including core 1 or T antigen (Galβ1-3GalNAcα-Ser/Thr), core 2 (GlcNAcβ1- 6[Galβ1-3]GalNAcα-Ser/Thr), core 3 (GlcNAcβ1-3GalNAcα-Ser/Thr), core 4 (GlcNAcβ1-6[GlcNAcβ1-3)GalNAcα-Ser/Thr), core 5 (GalNAcα1-3GalNAcα-Ser/Thr), core 6 (GlcNAcβ1-6GalNAcα-Ser/Thr), and core 8 (Galα1-3 GalNAcα-Ser/Thr), are also commonly linked to human proteins. These core structures can undergo further extension and branching to form more highly complex O-glycans [13].

Mucin type O-linked glycans are synthesized in the Golgi apparatus [14] in a stepwise and consensus motif-independent manner. A family of UDP-GalNAc polypeptide:N-acetyl-α-galactosaminyltransferases (GalNAc-Ts, EC 2.4.1.41) initiates the process by transferring N-acetylgalactosamine (GalNAc) from the charged sugar donor, UDP-GalNAc, to the hydroxyl group of serine and threonine amino acid residues (GalNAcα1-O-Ser/Thr). Abundance of GalNAc-T isoforms (at least 24 in humans [15] and 18 in mice [16]), distinct spatial and temporal gene expression patterns [16] and unique substrate specificities [17-21] ensure precise selection of attachment sites and fine regulation of GalNAc glycosylation of fully folded proteins. Depending on the final O-glycan structure, different sets of glycosyltransferases participate in elongation of the initial GalNAc residue. For example, UDP-Gal:GalNAcα-Ser/Thr β1-3 galactosyltransferase and UDP-GlcNAc:GalNAcα-Ser/Thr β1-3 *N*-acetylglucosaminyltransferase are required for synthesis of core 1 O-glycans and core 3 O-glycans, respectively.

A previous study reported attachment of core 1 Galβ1-3GalNAc disaccharide to rice glutelin basic subunits [22], however our *in silico *analysis and more recent experimental data [23] suggest that plants do not have the enzyme machinery to support mucin type O-glycosylation. In light of this, we reasoned that genetic manipulations could potentially modify plants for production of mammalian glycoproteins with homogenous O-glycan structures.

The present study tested, for the first time, the feasibility of generating a plant-based platform for production of Tn antigen-containing proteins/peptides. Many valuable pharmaceuticals, such as immunomodulators and tumor-associated antigens essential for the development of anticancer therapeutic vaccines, are GalNAc -glycosylated [24,25]. Despite a growing demand for these products, manufacturing remains difficult and costly. In our study, we first addressed the question of whether the initiation of mucin type O-glycosylation in plants could be performed with the precision typical of mammalian cells. Therefore, one of the well characterized isoforms of the human GalNAc-Ts family, GalNAc-T2, was stably expressed in *N. benthamiana *and used to demonstrate that mammalian GalNAc-Ts retain their functionality, specificity and localization in plant cells. We further tested the engineered GalNAc-T2 transgenic plants for their ability to produce GalNAc-glycosylated proteins. A model glycoprotein, mucosally targeted mucin 1 (MUC1) tandem repeat-derived peptide, was transiently expressed using the magnICON deconstructed viral vector system to ensure rapid accumulation and high yield [26]. Glycosylation efficiency was monitored by lectin chromatography. Our results suggest that co-expression of genes for UDP-GalNAc polypeptide:N-acetyl-α-galactosaminyltransferase, UDP-GlcNAc 4-epimerase, and UDP-GalNAc transporter are required to achieve efficient plant-based production of GalNAc-glycosylated proteins.

## Results

### Generation and molecular analyses of N. benthamiana L. plants expressing human GalNAc-T2

**Figure 1:**
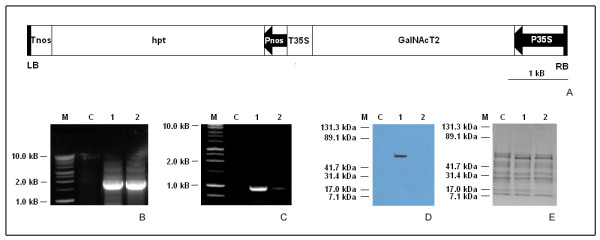
**Representative molecular analyses of *N. benthamiana *L. GalNAc-T2 transgenic plants**. **A **- Schematic diagram of T-DNA of pH7WG2:GNT2 plant binary expression vector. Abbreviations: LB, left border; RB, right border; Pnos, nopaline synthase promoter; P35 S, CaMV 35 S promoter; hpt, hygromycin phosphotransferase gene; Tnos, 3' termination signal of nopaline synthase gene; T35 S, 3' termination signal of CaMV 35 S gene; **B **- PCR screen using 1 μg genomic DNA as a template and primers G1/G2 (see "Materials and Methods") amplifying human GalNAc-T2 gene (1.7 kb); **C **- RT-PCR using 0.5 μg total RNA as a template and primers G3/G4 (see "Materials and Methods) that amplify a 0.96 kb fragment of the GalNAc-T2 transcript; **D **- Western blot of total hydrophobic proteins probed with antibodies raised against human GalNAc-T2; **E **- The same protein samples separated by SDS-PAGE (4-20% gradient gel) after staining with Coomassie Brilliant Blue R-250; Abbreviations used: C - control plant; 1,2 - putative transgenic plants; M- markers: Promega 1 kb DNA ladder (**B**, **C**), BioRad Kaleidoscope prestained standards (**D**, **E**).

*Agrobacterium*-mediated transformation of *N. benthamiana *L. with pH7WG2:GNT2 binary vector (Fig. 1A) was performed to generate plants stably expressing human GalNAc-T2 gene. The putative transgenic plants were subjected to routine molecular analyses, including PCR, RT-PCR and Western blotting, to select for the best producers. Plants that exhibited the expected PCR product of 1.7 kb in size (Fig. 1B), RT-PCR band of 0.96 kb in size (Fig. 1C), and a polypeptide of ~ 65 kDa after probing with monoclonal antibodies raised against the full length GalNAc-T2 protein (Fig. 1D) were selected for further experiments. None of the transgenic plants had apparent morphological abnormalities (data not shown).

### Localization of human GalNAc-T2 expressed in N. benthamiana L

**Figure 2:**
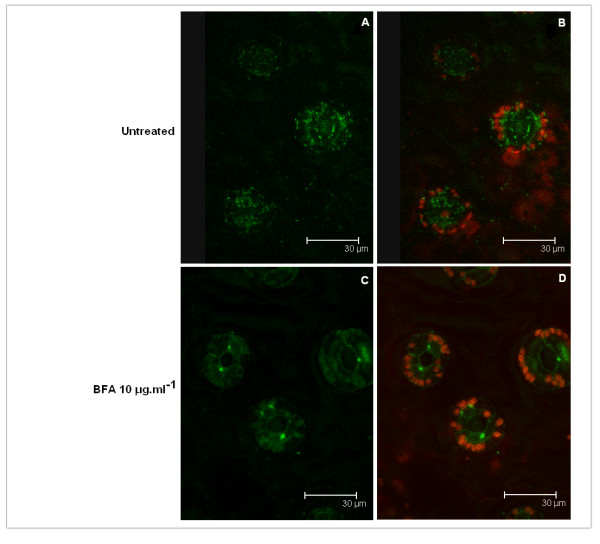
**Localization of human GalNAc-T2 expressed in *N. benthamiana *L. by confocal microscopy**. Image of leaf (guard cells) stably expressing GalNAc-T2:GFP fusion protein: **A **- image without chloroplasts, **B **- image with chloroplasts. Image of leaf (guard cells) stably expressing GalNAc-T2:GFP fusion protein after 1 h treatment with 10 μg/ml Brefeldin A: **C **- image without chloroplasts, **D **- image with chloroplasts. The green signal represents fluorescence of GalNAc-T2:GFP fusion protein, the red signal represents the autofluorescence of chloroplasts.

A fusion with *Aequorea victoria *green fluorescent protein (GFP), positioned at the C terminus of GalNAc-T2 to avoid interfering with the transmembrane domain (TMD), was used to monitor the localization of mammalian enzyme in tobacco cells. A discrete punctuate pattern was observed by confocal microscopy (Fig. 2A, B). This pattern could be indicative of Golgi localization or for targeting to the plant prevacuolar or late endosomal compartments (PVCs), as shown by experiments with tobacco Bright Yellow -2 cells expressing fluorescent markers [27]. To better define the localization of the expressed human GalNAc-T2, plant leaves were treated with Brefeldin A. Brefeldin A is a macrocyclic lactone isolated from *Alternaria carthami *known to block protein ER to Golgi transport. In plants, the drug causes loss of Golgi-based vesicle formation and fusion of the individual Golgi cisternae with the ER [28]. It also affects PVCs morphology leading to extensive aggregation and tubulation. However, in contrast to the high sensitivity of the Golgi apparatus, PVCs respond to Brefeldin A treatment only at drug concentrations greater than 50 μg/ml [27].

Tobacco plant leaves expressing GalNAc-T2:GFP fusion protein were incubated for 1 h with 10 μg/ml Brefeldin A. The treatment caused loss of the bright green spots and replacement of the punctuate pattern with a network-type fluorescence (Fig. 2C, D). These results strongly suggest that mammalian GalNAc-T2 is retained in the Golgi in plant cells.

### Functionality of human GalNAc-T2 expressed in N. benthamiana L

**Figure 3:**
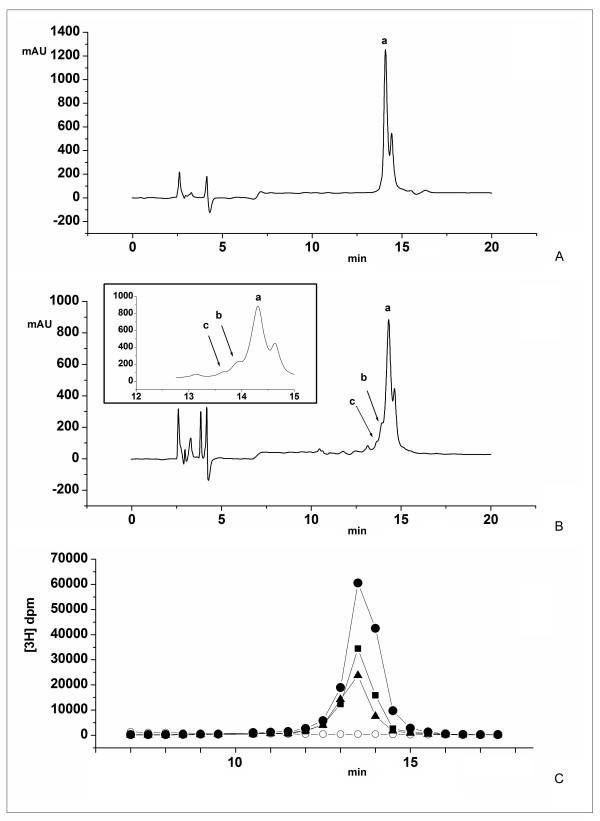
**Functional analyses of human GalNAc-T2 expressed *in N. benthamiana L*. plants**. GalNAc-T2 enzyme assay was set up in a final volume of 100 μl with UDP-GalNAc as the donor, and the hCG-β peptide as an acceptor (see "Materials and Methods"). A sample corresponding to 25 μl of the reaction mixture containing microsomal fraction of **A **- control plants, or **B **- GalNAc-T2 transgenic plants as an enzyme source, was subjected to RP-HPLC as described (see "Materials and Methods"): a - peak corresponding to the hCG-β peptide, b, c - peaks corresponding to the glycosylated forms of hCG-β peptide; **C **- monitoring the attachment of GalNAc to the hCG-β peptide by radioactive enzyme assay using [3H]UDP-GalNAc as a donor and 41 μg (●),, 20.5 μg (■) or 10.25 μg (▲) microsomal proteins from GalNAc-T2 plant, or 41 μg microsomal proteins from control plant (○) as an enzyme source. Aliquots of 0.5 ml from the collected fractions (2 ml fraction volume) were used to monitor the radioactivity.

**Figure 4:**
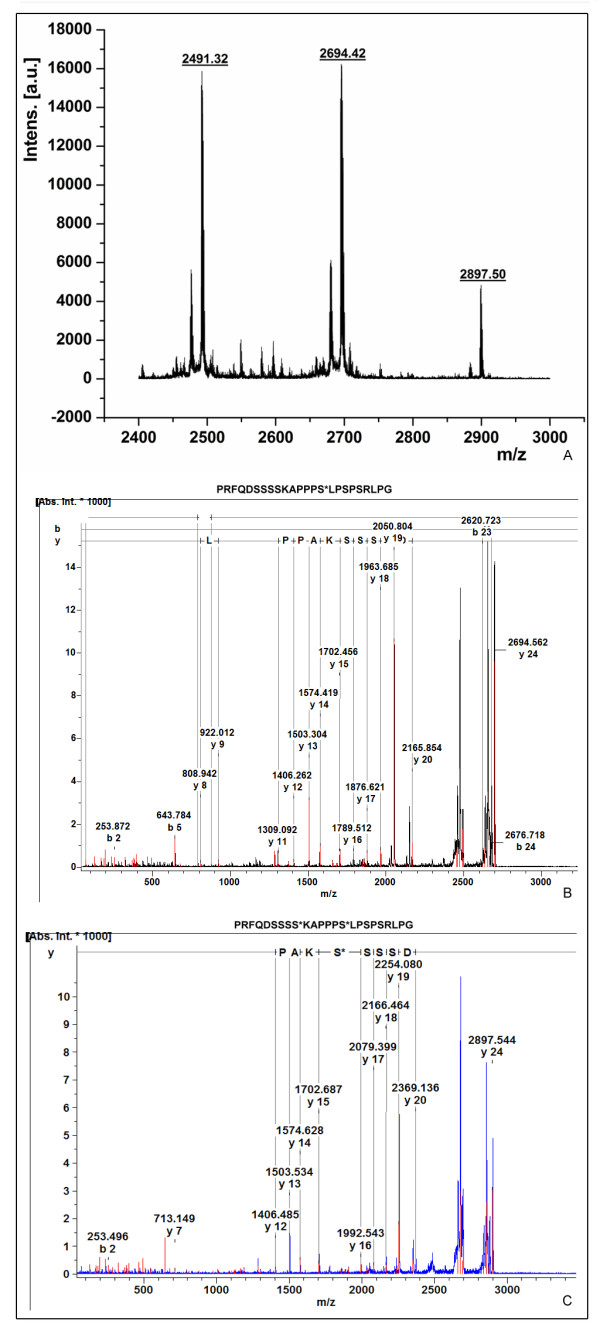
**Functional analyses of human GalNAc-T2 expressed *in N. benthamiana L*. plants**. **A **- MALDI-TOF MS of glycosylated products (peaks b + c combined) of hCG-β peptide; **B **- MALDI-TOF/TOF MS spectrum of [M+H+] 2694.4 corresponding to the monoglycosylated hCG-β peptide - GalNAc is attached to Ser-15 (corresponding to Ser-127 of hCG-β); **C **- MALDI-TOF/TOF MS spectrum of [M+H+] 2897.5 corresponding to the diglycosylated hCG-β peptide - GalNAc is attached to Ser-9 and Ser-15 (corresponding to Ser-121 and Ser-127 of hCG-β, respectively).

To assess the functionality of the plant-expressed human GalNAc-T2, we used an *in vitro *assay that included plant microsomal fraction as an enzyme source, UDP-GalNAc as the donor, and the 113-136 aa fragment of human chorionic gonadotropin β-subunit (hCG-β) as an acceptor peptide. Under the chosen HPLC conditions, the retention time for UDP-GalNAc was 4.09 min, while hCG-β peptide eluted at 14.08/14.43 min as two peaks ("a" and "a' "), as confirmed by MALDI (data not shown). HPLC separation of an aliquot of the enzymatic reaction with the microsomal fraction from control tobacco plants showed the presence of only the non-glycosylated hCG-β peptide (Fig. 3A). The observation was further confirmed by MALDI analysis (data not shown). In contrast, when microsomal fraction of GalNAc-T2 transgenic plants was used as the enzyme source, two additional peaks ("b" and "c") with slightly shorter retention times were visible on the chromatogram (Fig. 3B). Correlation between the accumulated radioactive label in the fractions corresponding to peaks "b" and "c" and the amount of enzyme added to the reaction mixture suggested that these peaks represent products of the enzymatic reaction (Fig. 3C). Further MALDI-TOF MS analysis of the combined "b" and "c" fractions revealed three major [M+H]^+ ^ions at *m/z *2491.3, 2694.4, and 2897.5 (Fig. 4A). The ion at *m/z *2491.3 indicated that the analyzed sample contained the initial (non-glycosylated) acceptor peptide. This was not surprising since the peak of the acceptor hCG-β peptide (peak "a") partially overlapped peaks "b" and "c". The ions at *m/z *2694.4 and *m/z *2897.5 were suggestive of two glycosylated forms of hCG-β peptide with attached one or two GalNAc residues. MALDI-TOF-TOF spectra analysis further supported the assumption. The major fragmentation product resulting from loss of one HexNAc residue from the glycosylated peptide containing a single GalNAc was evidenced by the abundant ion at *m/z *2491.4 (Fig. 4B). Similarly, during the fragmentation of the ion at *m/z *2897.5 corresponding to the hCG-β peptide with two GalNAc residues, the massive peak envelope centered at *m/z *2694.4 and smaller peak envelope at *m/z *2491.3 are indicative of the loss of one and two HexNAc residues, respectively (Fig. 4C).

*De-novo *peptide sequencing on MALDI-TOF/TOF MS spectrometer was employed to determine the position of the attached GalNAc residues. Based on b- and y-ions generated during the sequencing, the attachment of GalNAc for the mono- and diglycosylated peptides most likely occurred at Ser-15 (corresponding to Ser-127 of hCG-β; Fig. 4B), and at Ser-9 (corresponding to Ser-121 of hCG-β) and Ser-15 (Fig. 4C), respectively.

### Effect of the expression of human GalNAc-T2 on glycosylation status of N. benthamiana L. endogenous proteins

**Figure 5:**
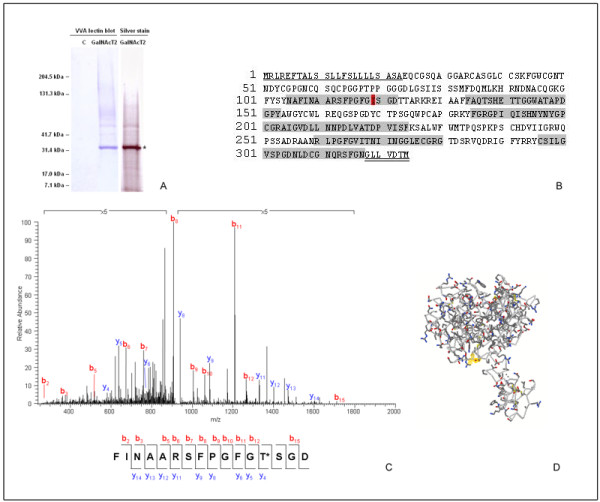
**Glycosylation of endogenous *N. benthamiana L***. proteins upon ectopic expression of human GalNAc-T2. **A **- VVA-lectin blot of the soluble proteins bound to VVA-agarose from control (C) and transgenic (GalNAc-T2) plants, and the corresponding silver staining for the GalNAc-T2 plant sample; **B **- sequence of tobacco endochitinase (GenBank Accession No P24091) identified with the 33 kDa band denoted in **A **by an asterisk: the signal peptide is underlined, the propeptide is double underlined, the covered regions after LC-MS/MS analysis of the chymotryptic fragments are gray-highlighted, the modified Thr-119 by 203 amu is red-highlighted; **C **- MS/MS analysis confirming the attachment of GalNAc (203 amu) to Thr-119; **D **- homology model of tobacco endochitinase built based on the 3 D structure of *O. sativa *L. japonica class I chitinase (pdb ID 2DKV); the position of the glycosylated Thr-119 is denoted by halos.

The substrate preferences of GalNAc-T2 have been extensively studied and the most favorable amino acids at positions -3, -2, -1, 1, 2, and 3 relative to the site of glycosylation (Ser/Thr), have been determined [29]. The first three amino acids with the highest enhancement values for each of these positions were used to design a series of peptides consisting of 7 amino acids. *In silico *analyses using the peptides to search NCBI plant databases (http://www.ncbi.nlm.nih.gov) indicated that some annotated plant proteins known to transport through the ER-Golgi pathway do contain potential sites for GalNAc-glycosylation (data not shown). Therefore, we tested for possible attachment of GalNAc to endogenous proteins of *N. benthamiana *L plants engineered to express human GalNAc-T2. Equal amounts of soluble proteins from the control and transgenic plants were subjected to affinity chromatography on *Vicia villosa *agglutinin (VVA)-agarose. VVA is a lectin that strongly recognizes terminal GalNAc residues attached to serine or threonine in a polypeptide. The binding buffer was supplemented with 0.1 M NaCl to prevent non-specific interactions, and the resin was thoroughly washed before the elution. Eluted proteins were further subjected to lectin blot analysis with alkaline phosphatase-conjugated VVA. Several positive bands were detected in the transgenic plant sample, but not in the negative control (Fig.5A). Following silver staining, the strongest band, corresponding to a 33 kDa polypeptide (Fig. 5A, denoted by an asterisk), was digested with chymotrypsin. Analysis of the recovered peptides by nano LC-MS/MS using a linear quadrupole ion trap mass spectrometer and a database search identified the band with 99% probability as *Nicotiana *endochitinase (GenBank Accession No P24091; EC 3.2.1.14; - Fig. 5B, Table 1). Modification of threonine at position 119 by 203 amu was also detected (Fig. 5C, Table 1), as evidenced by a series of y ions (y_4 _through y_14 _) containing the modification, thus strongly suggesting attachment of a N-acetylhexosamine residue to the polypeptide chain. It is of interest that cleavage on the carboxy-terminal side of the potentially modified Thr-119 was absent (lack of y_3 _and b_13 _ions), possibly as a result of the sugar moiety blocking access of the ionizing mobile proton at that position. Loss of the sugar moiety from glycopeptides is often observed during MS/MS, but in certain cases of low-energy-collision induced dissociation the sugar moiety may remain attached, resulting in fragment ions carrying the modification [30].

**Table 1:** LC-MS/MS analysis of in-gel chymotrypsin-digested *N. benthamiana *L. 33 kDa endogenous polypeptide band identified as endochitinase (EC 3.2.1.14, GenBank Accession No P24091).

Peptide sequence	MH+	Xcorr	dCn
(Y)C*SILGVSPGDNLDC*GNQRSF(G)	2197.29	4.24	0.12

(F)FAQTSHETTGGWATAPDGPY(A)	2095.17	3.98	0.77

(L)NNPDLVATDPVISF(K)	1502.65	3.68	0.79

(F)AQTSHETTGGWATAPDGPY(A)	1948.00	3.56	0.85

(L)GVSPGDNLDC*GNQRSFGN(G)	1894.91	3.54	0.44

(F)FAQTSHETTGGW(A)	1322.37	3.48	0.85

(L)GVSPGDNLDC*GNQRSF(G)	1723.76	3.30	0.31

(Y)NYGPC*GRAIGVDLL(N)	1505.67	3.19	0.60

(Y)NAFINAARSFPGFG**T**SGD(T)	2032.95	2.94	0.73

(F)INAARSFPGF(G)	1080.22	2.80	0.60

(N)RLPGFGVITN(I)	1074.26	2.69	0.85

(F)GRGPIQISHNY(N)	1242.37	2.67	0.79

(L)LNNPDLVATDPVISF(K)	1615.81	2.63	0.69

(Y)FGRGPIQISHNY(N)	1389.54	2.57	0.85

(V)ITNIINGGLEC*GRG(T)	1474.62	2.51	0.78

(A)FINAARSFPGFG**T**SGD(T)	1847.77	2.37	0.65

(F)INAARSFPGFG**T**SGD(T)	1700.59	2.13	0.84

The threonine residue (Thr-119) modified by 203 amu is in boldface type, carboxymethylated cysteine residues are denoted by asterisks. The amino acids in parenthesis at the beginning and end of each peptide show the flanking residue in the entire protein sequence.

A homology model of tobacco endochitinase (Fig. 5D), built based on the three dimensional structure of *O. sativa *L. japonica class I chitinase (pdb ID 2DKV, 66. 1% identity), suggests that Thr-119 should be located on the surface of the catalytic domain, therefore accessible for glycosylation. Previous study has shown that Thr-119 is not directly involved in the catalytic act [31].

### Efficiency of attachment of GalNAc to the model protein, E. coli enterotoxin B subunit:human MUC1 tandem repeat-derived peptide fusion protein expressed transiently in GalNAc-T2 plants by magnICON deconstructed viral vector system

**Figure 6:**
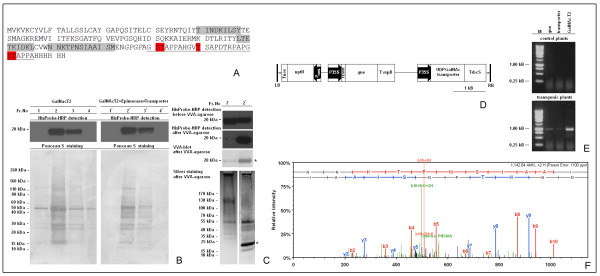
**Characterization of LTBMUC1 produced in transgenic *N. benthamiana *L. plants expressing either human GalNAc-T2 or co-expressing human GalNAc-T2, *Y. enterocolitica *UDP-GlcNAc-4 epimerase, and *C. elegans *UDPGlcNAc/UDPGalNAc transporter**. **A **- amino acid sequence of His-tagged LTBMUC1 fusion protein: MUC1 sequence is underlined, GPGP linker is italisized, experimentally confirmed sites for attachment of GalNAc by GalNAc-T2 are red-highlighted, covered regions after LC-MS/MS analysis of the chymotryptic fragments are gray-highlighted; **B **- purification of LTBMUC1 produced in GalNAc-T2 and GalNAc-T2//UDP-GlcNAc-4 epimerase//UDP-GlcNAc/UPD-GalNAc transporter plants by Ni^2+ ^Sepharose Fast Flow chromatography: Fractions (1, 2, 3, 4 for LTBMUC1 produced in GalNAc-T2 plants; and 1', 2', 3', 4' for LTBMUC1 produced in the triple transgenic plants) eluted from the resin with 20 mM phosphate buffer pH 7.4 containing 0.5 M NaCl and 0.5 M imidazol, were screened by HisProbe-HRP (upper panels); the same blots were stained in parallel with Ponceau S (lower panels); **C **- VVA chromatography of LTBMUC1 produced in the tested plant systems after purification on Ni^2+ ^Sepharose Fast Flow: identical aliquots of the volume-equalized samples 2 and 2' were screened by HisProbe-HRP before loading on VVA agarose gel (panel HisProbe-HRP detection before VVA agarose); bound proteins were eluted from the resin with 50 mM Tris HCl buffer pH 7.4 containing 0.1 M NaCl, 1 mM MgCl_2 _and 0.3 M GalNAc, and identical aliquots were screened by HisProbe-HRP (panel HisProbe-HRP detection after VVA agarose), VVA blotting (panel VVA blot after VVA agarose), and SDS-PAGE with subsequent silver-staining (panel silver staining after VVA agarose); asterisk denotes the position of LTBMUC1; **D **- schematic diagram of T-DNA of pBYR:GNE.GT plant binary expression vector. Abbreviations: LB, left border; RB, right border; Pnos, nopaline synthase promoter; P35 S, CaMV 35 S promoter; nptII, neomycin phosphotransferase gene; gne - *Y. enterocolitica *UDP-GlcNAc-4-epimerase gene; UDP-GalNAc - *C. elegans *UDP-GlcNAc/UDP-GalNAc transporter gene; Tnos, 3' termination signal of nopaline synthase gene; T35 S, 3' termination signal of CaMV 35 S gene; TvspB - 3' termination signal of soybean vegetative storage protein B gene; TrbcS - 3' termination signal of Rubisco small subunit gene; TEV HT - etch virus translational enhancer element; **E - **RT-PCR confirmation of the co-expression of human GalNAc-T2 (GalNAc-T2), *C. elegans *UDP-GlcNAc/UPD-GalNAc transporter (transporter), and *Y. enetrocolitica *UDP-GlcNAc-4 epimerase (gne) in *N. benthamana *L. plants using 0.5 μg total RNA as a template and primers G3/G4, GT3/GT4, and GE4/GE5, respectively (see "Materials and Methods). **F **- spectrum of a chymotryptic peptide of the 20/21 kDa band denoted in **C **by an asterisk, assisting in the identification of the band as LTBMUC1.

As the transgenic GalNAc-T2 plants demonstrated capability for attaching GalNAc to the endogenous endochitinase, we further examined their potential to glycosylate a model protein expressed at high levels. A synthetic gene coding for a C-terminal His-tagged fusion between the *E. coli *enterotoxin B subunit and human MUC1 tandem repeat (TR)-derived peptide (LTBMUC1) was used for this purpose (Fig. 6A). Mucins are heavily glycosylated membrane-bound proteins containing serine and threonine-rich TRs that are often polymorphic and vary in number [32]. In most cancers of glandular epithelial origin, MUC1 is overexpressed and aberrantly glycosylated, leading to exposure of highly immunogenic truncated carbohydrate structures [33]. Tn antigen is one of the most common among them, and some studies have already demonstrated the potential of MUC-1 TR-derived GalNAc-glycopeptides for active immunotherapy of cancer patients [34,35].

The MUC1 TR-derived peptide used as a part of the model LTBMUC1 protein has seven potential O-glycosylation sites predicted *in silico *by NetOGlyc (Ser-131, Thr-132, Thr-140, Ser-141, Thr-145, Ser-151, and Thr-152), but only serine and threonine in GSTAP motif (Ser-131, Thr-132, Ser-151, and Thr-152) and threonine in GVTSA motif (Thr-140) have been verified experimentally as target sites of GalNAc-T2 [36], (Fig. 6A). Therefore, upon a complete GalNAc-glycosylation by GalNAc-T2, the molecular mass of the recombinant protein should increase by ~ 1 kDa, which is difficult to resolve during electrophoresis to differentiate the glycosylated and non-glycosylated forms. However, the forms can be distinguished by exploiting differences in their affinity to a GalNAc-specific lectin. Accordingly, the strategy to verify LTBMUC1 glycosylation was designed as follows: (i) transient expression of the model protein using the magnICON deconstructed viral vector system to ensure rapid accumulation and high yield [26]; (ii) purification by Ni^2+ ^Sepharose Fast Flow chromatography combined with a His tag-based screen to detect the fractions containing the recombinant protein; (iii) affinity chromatography on VVA agarose to capture the GalNAc-glycosylated population of LTBMUC1, assisted by a His-tag-based and VVA-lectin blot detection to verify the molecular mass of the captured protein; (iv) LC-MS/MS analysis to confirm the identity of LTBMUC1 and determine the sites of glycosylation.

Leaves of the GalNAc-T2 transgenic tobacco plants were transiently transformed with the magnICON 3' vector module pICH11599:LTBMUC1 co-delivered with the 5' module pICH15879 and the integrase module pICH14011 [26]. Because of rapidly developed necrosis leaves were harvested relatively early at 7 day post-infiltration. The levels of LTBMUC1 at that time were 3% of the total soluble proteins at most (Pinkhasov *et al*., submitted). Following protein extraction and ammonium sulfate precipitation, the His-tagged recombinant protein was purified by chromatography on Ni^2+ ^Fast Flow Sepharose. Aliquots of the fractions eluted from the column were analyzed for purity by Ponceau S staining and by Western blotting to detect the His-tag on LTBMUC1. The recombinant protein was not visible on the Ponceau S-stained PVDF membranes, probably because its amount was below the detection limit of the stain (1 μg/5 mm band). It should be noted that the LTBMUC1-positive fractions contained co-eluted endogenous tobacco proteins (Fig. 6B, left side, lower panel). The main contaminant appears to be the enzyme ribulose-1,5-bisphosphate carboxylase/oxygenase represented under denatured conditions by its large (~ 53 kDa) and small (~15 kDa) subunits. The protein is known to be a major obstacle in achieving homogenous preparations of plant-expressed biopharmaceuticals [37].

The theoretical molecular mass of LTBMUC1 is 17.7 kDa, but on the His-tag blots the recombinant protein migrated as a single ~20 kDa band (Fig. 6B, left side, upper panel). An ~20 kDa band was also detected upon expression of LTBMUC1 in control, nontransgenic *N. benthamiana *L. plants; this band was positively stained with Roche Diagnostics DIG Glycan Detection Kit (Pinkhasov et al., submitted). These data suggest that plant-expressed LTBMUC1 undergoes glycosylation by endogenous enzyme activities. The amino acid sequence of LTBMUC1 lacks consensus motifs for N-glycosylation (Asn-Xaa-Ser/Thr), but there are several proline residues in a context favoring their hydroxylation and subsequent glycosylation [7-9]. Therefore, it is likely that plant-expressed LTBMUC1 is decorated by plant-specific O-glycans, arabinogalactans and/or arabinans.

Lectin chromatography on VVA agarose coupled with VVA lectin blot and His-tag-assisted detection further demonstrated that trace amounts of LTBMUC1 expressed in GalNAc-T2 transgenic plants were modified by GalNAc-glycosylation (Fig. 6C, left side).

### Co-expression of GalNAc-T2, UDP-GlcNAc-4 epimerase and UDP-GalNAc transporter is required for GalNAc-glycosylation of recombinant proteins expressed in N. benthamiana L. at high levels

The activity of GalNAc-T is not the only factor affecting the rate of GalNAc-transfer to serine/threonine residues on acceptor proteins. The concentration of the UDP-GalNAc donor and the efficiency of transfer to the Golgi lumen also contribute to the final outcome. Thus, we further tested whether a boost in the endogenous UDP-GalNAc substrate pool or the translocation of UDP-GalNAc to the Golgi lumen could improve the efficiency of LTBMUC1 GalNAc-glycosylation. GalNAc-T2 plants were subjected to another round of transformation with either the *Y. enterocolitica gne *gene coding for the enzyme UDP-GlcNAc 4-epimerase that catalyzes the epimerization of UDP-GlcNAc to UDP-GalNAc, or the *C. elegans *C03H5.2 gene encoding a protein capable of simultaneous and independent transport of UDP-GlcNAc and UDP-GalNAc [38]. The double transgenics were further used for production of LTBMUC1 as described earlier, and in both cases the efficiency of glycosylation of the recombinant protein showed no improvement (data not shown). Next, *Y. enterocolitica gne *gene and *C. elegans *C03H5.2 gene were delivered simultaneously by pBYR:GNE.GT plant binary vector (Fig. 6D) and expressed successfully in GalNAc-T2 plants as evidenced by the RT-PCR analysis (Fig. 6E). The model LTBMUC1 protein expressed in the triple transgenics and purified on Ni^2+ ^Fast Flow Sepharose (Fig. 6B, right side), was completely retained on the VVA agarose column, which indicated that it was present exclusively as a GalNAc-glycosylated form.

An outline of the analyses of LTBMUC1 expressed in GalNAc-T2 plants (2) and GalNAc-T2//UDP-GlcNAc 4-epimerase//UDP-GlcNAc/UDP-GalNAc transporter plants (2') after Ni^2+ ^Fast Flow Sepharose chromatography is presented in Fig. 6C. Identical aliquots of volume-equalized samples were run in parallel and assayed (i) before VVA chromatography: by His-tag blotting to demonstrate that comparable amounts of LTBMUC1 (2 and 2') were loaded on the columns (panel "Hisprobe-HRP detection before VVA agarose"), and (ii) after elution of the bound proteins from VVA agarose resin: by VVA lectin blotting to confirm the attachment of GalNAc to the polypeptide acceptors (panel "VVA blot after VVA agarose), and by His-tag blotting to determine the portion of the loaded LTBMUC1 with affinity to the VVA lectin (panel "Hisprobe-HRP detection after VVA agarose). In contrast to LTBMUC1 expressed in GalNAc-T2 plants, the recombinant protein produced in the triple transgenics showed His-tag signals of comparable intensity before and after passage through the lectin column and a strong band upon detection by VVA lectin blotting. In addition, a slight delay in its electrophoretic mobility was detected (panel "Hisprobe-HRP detection before VVA agarose). These results clearly indicate that efficient GalNAc-glycosylation of LTBMUC1 was achieved only upon expression of the model protein in the GalNAc-T2//UDP-GlcNAc 4-epimerase//UDP-GlcNAc/UDP-GalNAc transporter plants.

Bound proteins that eluted from the VVA-agarose column from both LTBMUC1 samples were analyzed by SDS-PAGE and subsequent silver staining. A strong band with a molecular mass of ~20/21 kDa was visible only in the sample from the triple transgenics (Fig. 6C, right side, denoted by an asterisk). The band was identified as LTBMUC1 by chymotryptic digest coupled with nano LC-MS/MS analysis (Table 2, Fig. 6A, F). Peptides from the MUC1 region of the recombinant protein were not recovered, even after alternative in-gel digestion with other proteinases (trypsin, GluC). Thus, this prevented identification of the GalNAc attachment sites.

**Table 2:** LC-MS/MS analysis of in-gel-chymotrypsin-digested LTBMUC1 produced in *N. benthamiana *L. plants co-expressing human GalNAc-T2, *Y. enterocolitica *UDP-GlcNAc-4 epimerase and *C. elegans *UDP-GlcNAc/UDP-GalNAc transporter.

Peptide sequence	MH+	Xcorr	dCn
(Y)LTETKIDKL(C)	1060.80	2.69	0.61

(W)NNKTPNSIAAI(S)	1142.84	2.61	0.84

(W)NNKTPNSIAAISM(S)	1361.75	2.37	0.64

(Y)TINDKILSY(T)	1066.36	1.99	0.73

The amino acids in parenthesis at the beginning and end of each peptide show the flanking residue in the entire protein sequence.

## Discussion

GalNAc-T2 is one of the best characterized members of the GalNAc-Ts family that exhibits a very low level of UDP-GalNAc hydrolase activity [29]. Thus, it was chosen for a proof of concept study to determine if mammalian enzymes could be engineered into plants capable of initiating mucin type O-glycosylation. Our work provides experimental evidence for several conclusions. Based on the detected single, sharply defined ~ 65 kDa band after Western blot analysis of the GalNAc-T2 transgenics, it appears that the human enzyme is stably expressed in plants and similar to expression in mammalian cells the consensus Asn-516/Xaa(Asp)-517^/^Ser-518 sequence remains unoccupied by N-glycans [39]. The human GalNAc-T2 appears to retain its Golgi localization in tobacco cells as evidenced by the GFP-tagging and alteration of the fluorescence pattern after treatment with Brefeldin A. The latter result corroborates the idea that some targeting signals and/or targeting mechanisms might be conserved between plants and animals [40]. The plant-expressed human GalNAc-T2 enzyme is functional and retains its specificity, as demonstrated by its ability to glycosylate the 113-136 aa fragment of hCG-β defined as a GalNAc-T2-specific acceptor [36] at sites identical to the glycosylation sites of hCG hormone produced by placental trophoblasts in late pregnancy [41,42]. Specifically, the attachment of GalNAc to the hCG-β peptide occurs at serine residues corresponding to Ser-121 and Ser-127 of the hCG-β subunit. Our results also suggest that attachment of GalNAc to Ser-127 precedes the attachment of GalNAc to Ser-121. To our knowledge, there are no previous studies addressing the pathway of incorporation of GalNAc in hCG or hCG-β-derived peptides. However, the work of Sugahara *et al*. [43] supports our conclusion, having demonstrated that: (i) Ser-127 defines the carboxy border of Ser-121 recognition site; (ii) the context requirements for Ser-127 to act as an acceptor site are restricted to the presence of Pro-131 and Ser-132, and do not include Ser-121; (iii) elimination of Ser-121 by site-directed mutagenesis does not impair the acceptor potential of Ser-127. In addition, in contrast to Ser-121, Ser-127 is N-terminally flanked by an adjacent proline residue which, according to a model study with a library of oriented random peptides, defines Ser-127 as the more favorable site for glycosylation by GalNAc-T2 [29].

*In silico *analysis suggests that plants do not have orthologs of human GalNAc-Ts. By a combination of *in vitro *radioactive enzyme assay and mass-spectrometry analysis, our work provides direct experimental evidence for a lack of endogenous GalNAc-T2 activity in *N. benthamiana *L. plants. A similar conclusion was conveyed indirectly by a study showing absence of mucin type O-glycans in the hinge region of maize-produced human IgA1 [23]. Our work further demonstrates that ectopic expression of human GalNAc-T is sufficient to "arm" plant cells with the ability to initiate mucin type O-glycosylation, as evidenced by the appearance of several endogenous proteins with affinity to the GalNAc-specific lectin, VVA. Furthermore, we provide MS evidence for the attachment of GalNAc to one of those proteins, the vacuole-targeted enzyme endochitinase that travels through the ER-Golgi route [31]. These data suggest that besides the enzyme entity, all other elements necessary for initiation of mucin type O-linked glycosylation, including a UDP-GalNAc donor and protein capable of translocating UDP-GalNAc from the cytosol into the Golgi lumen, exist in plant cells. The presence of free UDP-GalNAc in plants has not been documented so far, but a cytosolic enzyme, UDP-glucose 4-epimerase capable of converting UDP-GlcNAc, an abundant plant compound, to UDP-GalNAc, in addition to its primary substrate, UDP-glucose, has been identified [38]. A nucleotide sugar transporter with broader substrate specificity could deliver the activated GalNAc to the Golgi lumen. Such function is likely to be performed by still unknown or one (or more) of the identified plant UDP-galactose transporters [44-46]. Similarly to *H. sapiens *and *D. melanogaster *UDP-galactose transporters [47], the latter might have the ability to transport UDP-GalNAc in addition to their primary substrate UDP-galactose. However, the endogenous plant UDP-GalNAc pool and UDP-GalNAc transporter activity are apparently low and could be easily "exhausted" by endogenous substrates, as only a very small portion of the recombinant LTBMUC1 was subjected to glycosylation. In corroboration of this conclusion, co-expression of human GalNAc-T2 with *Y. enterocolitica *UDP-GlcNAc 4-epimerase and *C. elegans *UDP-GlcNAc/UDP-GalNAc transporter resulted in the production of LTBMUC1 exclusively as a glycoform. Altered electrophoretic mobility of the model protein, and especially its strong binding to VVA-agarose, are unequivocal proofs for attachment of GalNAc residue(s) to the polypeptide backbone.

Similar to previous observations with maize-expressed human IgA1 [23], tobacco-expressed LTBMUC1 also appears to be decorated by plant-specific glycans attached to hydroxyproline residues (Pinkhasov et al., submitted). This modification explains the discrepancy between the calculated (17.7 kDa) and the apparent (~20 kDa) molecular mass of the model protein. GalNAc-glycosylation of LTBMUC1 upon expression in GalNAc-T2//UDP-GlcNAc 4-epimerase//UDP-GlcNAc/UDP-GalNAc transporter plants decreases further the electrophoretic mobility of LTBMUC1. Although the shift appears to reflect the expected contribution of the GalNAc residues (~0.2 - ~1 kDa) to the protein mass, it is difficult to relate that shift to exact numbers of attached GalNAc residues. Our attempts to gain more information about the occupancy of the glycosylation sites failed because of the resistance of MUC1 to LC-MS/MS-compatible proteolytic cleavage. Therefore, it remains unclear if the presence of plant specific O-glycans "shielded" some of the GalNAc-attachment sites of the model protein used in this study. It is also unclear if these O-glycans might become a source of immunogenicity or affect the stability and/or the activity of plant-produced LTBMUC1. These general issues should be addressed for any plant-produced O-glycosylated protein therapeutic. A possible approach to eliminate the attachment of the most common plant-specific O-glycans, arabinans and arabinogalactans, to the recombinant pharmaceuticals, would be to prevent the conversion of proline to 4-hydroxyproline. The expansion of our knowledge about plant prolyl-4-hydroxylase family would allow targeted knockout to eliminate the activity of a certain member of the family, thereby preventing/minimizing detrimental effects on the plant development. Alteration of the amino acid sequence of the recombinant proteins targeted for plant-based expression that aims at elimination of motifs favoring prolyl hydroxylation [9] could serve as an alternative approach..

## Conclusion

Our work provides the first experimental evidence that plants can be engineered for production of Tn-antigen-containing therapeutics by "updating" their enzyme machinery with UDP-GlcNAc 4-epimerase, UDP-GalNAc transporter and GalNAc-T. Further genetic manipulations could potentially lead to the synthesis of more complex O-glycans. Thus, plants can become an attractive alternative platform for production of homogenous populations of mucin type O-glycosylated recombinant therapeutics with predictable O-glycan structures. Endogenous plant enzyme activities that target directly the actual sites of O-glycan attachment, serine and threonine residues, are not as abundant as in other heterologous production systems (e.g. O-mannosyltransferase activity in yeasts). However, proline residues are often in proximity to the attachment sites, and some of these prolines might be in a sequence context favoring their plant-specific hydroxylation and subsequent glycosylation. The possibility of "collision" between the plant O-glycosylation process and the engineered GalNAc-glycosylation process requires careful examination of the amino acid sequences of mammalian proteins targeted for plant-based expression, and if necessary and possible, appropriate alterations to eliminate the risky motifs.

## Methods

### Construction of plant binary expression vectors

The coding region of *Homo sapiens *GalNAc-T2 (GenBank Accession No BC041120) was amplified with Pfu DNA polymerase (Strategene, La Jolla, CA) using G1 (5'-CACCATGCGGCGGCGCTCGCGGATGC-3')/G2 (5'-CAGCTACTGCTGCAGGTTGAGCGTGAACTTCCACTGCTGCGAAAGGGCC-3') pair of primers. The product was separated on 1% agarose gel, gene-cleaned (QIAquick Gel Extraction, Qiagen, Valencia, CA) and subcloned in pENTR/D-TOPO vector (Invitrogen, Carlsbad, CA). Using the Gateway™ technology (Invitrogen, Carlsbad, CA), the gene was transferred either to pH7WG2, or to pK7FWG2 plant expression vector [48] for native or fusion GFP-tagged protein expression, respectively. The final constructs designated as pH7WG2:GNT2 and pK7FWG2:GNT2, were verified by sequencing (Applied Biosystems 377 gel sequencer, ASU), and transferred by electroporation to *A. tumefaciens *EHA101 competent cells. Recombinant colonies were selected on LB plates supplemented with 200 mg/l spectinomycin.

*Caenorhabditis elegans *clone (CO3H5.2) coding for UDPGlcNAc/UDPGalNAc transporter [38], was purchased from Open Biosystems (Huntsville, AL), and amplified using GT1 (5'-CA**CCATGG**ATCGAGCTAACGACACGAGCTC-3')/GT2 (C**GGTACC**TCAGGCATTATGAGCTTCGGCTG-3') set of primers. *Yersinia enterocolitica *type 0:8 *gne *gene coding for UDP-GlcNAc-4 epimerase was isolated by a PCR approach using GE1 (5'-CACC**TCTAGA**ATGTCTATATTAATTACTGGTGGTGCTG-3')/GE2 (5'-CC**GAGCTC**CTAACAGTTATAACCATTAGGATTCATTTTTTGCC-3') pair of primers. Both PCR products were subcloned in pENTR/D-TOPO vector (Invitrogen, Carlsbad, CA). The internal *Xba*I site of the *gne *gene was eliminated by a site-directed mutagenesis (QuickChange Multi Site-Directed Mutagenesis Kit, Stratagene, La Jolla, CA) using GE3 (5'-CTGAACTAGCACATC**G**AGAGTTAGGTTGGTATGC-3') primer. A shuttle pBY210:GNE:GT vector containing both *C. elegans *UDPGlcNAc/UDPGalNAc transporter gene and *Y. enterocolitica gne *gene was generated using a two-step strategy. First, the *gne *gene was ligated as an *Xba*I/*Sac*I fragment into pBYsE1bE2T210 (unpublished, H. Mason; details to be published elsewhere) digested with the same restriction enzymes. The generated vector was then cut open with *Nco*I and *Kpn*I for ligation of the transporter gene taken from pENTR/D:GT as an *Nco*I/*Kpn*I fragment. The cassette with the two genes was further excised with *Asc*I and *Fse*I, and ligated into *Asc*I/*Fse*I digested pBYR-H2gpKDEL-K3 (unpublished, H. Mason; details to be published elsewhere), thus producing pBYR:GNE.GT vector. The vector was electroporated into *A. tumefaciens *LB4404 competent cells. Recombinant colonies were selected in the presence of 200 mg/l streptomycin.

The construction of pICH11599:LTBMUC1 vector is described elsewhere (Pinkhasov *et al*., submitted). The vector is the 3' module of the magnICON deconstructed viral expression system (ICON Genetics, Halle, Germany), and is delivered simultaneously with the integrase module pICH14011 and the 5' module pICH15879. The system, based on the assembly of functional viral replicons *in planta*, is described in details in [26]. All module vectors were electroporated into *A. tumefaciens *GV3101 competent cells and selected in the presence of 50 mg/L carbenicillin and 60 mg/L rifampicin.

### Plant transformation

Stable *Agrobacterium*-mediated transformation of *N. benthamiana *L. plants was performed by a standard protocol [49]. Depending on the vector used, transformants were selected on MS medium [50] containing 50 mg/L hygromycin or 100 mg/L kanamycin, 500 mg/L cefotaxime, 1 mg/L BAP and 0.1 mg/L NAA. Developed shoots were transferred to a phytohormone-free MS medium containing 50 mg/L hygromycin or 100 mg/L kanamycin, and 300 mg/L cefotaxime for root formation. Regenerated plants were transferred from the Magenta boxes to pots, and further grown under greenhouse conditions (26°C, 16L/8D).

Transient transformation *in planta *was performed by a syringe infiltration method. The suspensions of *Agrobacterium *cells harboring the expression vectors were infiltrated in fully expanded leaves of wild type of transgenic *N. benthamiana *L. plants. Bacterial suspensions in 10 mM MES buffer pH 5.6 containing 10 mM MgSO_4 _were adjusted to either OD_600 _= 0.2 (for pBYR:GNE.GT), or OD_600 _= 0.033 (for each of the three magnICON module vectors). Infiltrated plants were kept in growth chambers (26°C, 16L/8D) until ready for leaf harvest.

### Molecular analyses of the transgenic plants

A PCR screen of the putative GalNAc-T2 transgenic plants was performed with GoTaq DNA Polymerase (Promega, Madison, WI), 1 μg genomic DNA (DNeasy Plant Mini Kit, Qiagen, Valencia, CA) as a template, and the same primers and PCR program used for GalNAc-T2 gene amplification.

Total RNA was isolated with Promega's SV Total RNA Isolation System. RT-PCR was performed using a TITANIUM One-Step RT-PCR Kit (BD Biosciences, Mountain View, CA), and 500 ng of template. The following pair of primers were used: G3 (5'-GGTGATCACGTTTCACAATGAAGCCAGG-3')/G4 (5'-AGTGTCGAGGCAGTTAGTTCCCTGCTGC-3') pair for amplification of a 0.96 kb fragment of the human GalNAc-T2 transcript, GT3 (5'-GACACGAGCTCCAATCTGAAGCTCATCTC-3')/GT4 (5'- CAGGCATTATGAGCTTCGGCTGGTGTCG-3') pair for amplification of a 1.04 kb of *C. elegans *UDP-GlcNAc/UPD-GalNAc transporter transcript, and GE4 5'-GTCTATATTAATTACTGGTGGTGCTGGATATATAGG-3')/GE5 (5'-CAGTTATAACCATTAGGATTCATTTTTTGCCATTTCCAAGCGTCC-3') pair for amplification of 1.07 kb of *Y. eneterocolitica gne *transcript. The PCR and RT-PCR samples were separated on agarose gels along with BenchTop 1 kb DNA ladder (Promega, Madison, WI) or GeneRuler 1 kb DNA ladder (Fermentas Inc., Glen Burnie, MD), and visualized by staining with ethidium bromide.

For Western blot analysis, the hydrophobic proteins from the transgenic and control plants' leaves were isolated with a Hydrophobic Protein Isolation Kit (Sigma, St. Louis, MO). SDS samples were prepared with 5×SDS sample buffer (0.2 M Tris HCl pH 6.8, 3.7 M β-mercaptoethanol, 10% (w/v) SDS, 0.05% (w/v) bromophenol blue) and loaded on 4-20% gradient gels (BioRad, Hercules, CA) next to Kaleidoscope Prestained Standards (BioRad, Hercules, CA). Separated proteins were electro-blotted onto 0.45 μm Immobilon-P PVDF membranes (Millipore, Billerica, MA) and blocked overnight at 4°C in TBST buffer (20 mM Tris HCl pH 7.4, 180 mM NaCl, 0.05% (v/v) Tween-20) containing 5% (w/v) dried milk. Following two washes with TBST for 15 minutes each, the membranes were probed overnight at 4°C with mouse monoclonal IgG antibodies (10 μg CB-4 in 5 ml TBST containing 1% (w/v) dried milk) raised against human GalNAc-T2 (CellMab AB, Sweden) as originally described in [51]. Next day, the blots were washed as described above, and incubated for 1 hour at room temperature with 1:3000 diluted anti-mouse horse radish peroxidase-conjugated antibodies. This was followed by four more washes (2 × 15 min in TBST and 2 × 5 min in double distilled water), and an ECL detection performed according to the manufacturer (Amersham Biosciences, Piscataway, MA).

### GalNAc-T2 enzyme assay

Plant leaves were homogenized with a mortar and pestle in cold 50 mM Tris HCl buffer pH 7.4 containing 330 mM sucrose, 3 mM DTT, 3 mM EDTA, 5% polyvinyl pyrrolidone and 1 mM PMSF. The homogenate was filtered through cheesecloth and centrifuged at 10 000 g for 25 minutes at 4°C. The supernatant was then centrifuged at 100 000 g for 2 hours (Beckman Coulter Optima L-100 XP Ultracentrifuge). The pellet representing microsomal membranes was resuspended in 50 mM Tris HCl pH 7.4, and protein concentration was measured according to the assay described in [52]. The GalNAc-T2 enzyme assay was performed at 37°C in 100 μl reaction mixture containing 25 mM Tris HCl buffer pH 7.4, 10-45 μg microsomal proteins, 500 μg acceptor peptide (the 113-136 aa fragment of human chorionic gonadotropin β-subunit, hCG-β, synthesized by the Proteomics and Protein Chemistry Lab, ASU and verified by mass spectrometry), 10 μg UDP-GalNAc or 1.5 μCi [3H]UDP-GalNAc (American Radiolabeled Chemicals, St. Louis, MO), 10 mM MnCl_2_, and 0.25% (v/v) Triton X-100. At the completion of the reaction, the mixture was filtered sequentially through Ultrafree-MC centrifugal filter (Millipore, Bedford, MA) and Nanosep 30 K filter spin columns (Pall Corporation, East Hills, NY).

### Identification of the reaction products by mass-spectrometry (MS) and peptide sequencing

An aliquot of the reaction mixture was subjected to reverse-phase HPLC (Agilent 1100 system, Waters, Milford, MA) using Luna column (C18, 5 μm, 10 × 250 mm; Phenomenex, Torrance, CA). The compounds were eluted at a constant flow rate of 4 ml/min with a linear (10-45%) gradient of solvent B (0.1% trifluoroacetic acid (TFA), 90% acetonitrile in water) in solvent A (0.1% TFA in water) for 20 minutes, followed by a linear gradient (45-95%) of solvent B in solvent A for 10 min. The elution of the peptides was monitored by diode-array detector at 214 nm. Fractions of 2 ml were collected throughout and monitored for incorporated radioactivity on a LS 6500 Multipurpose Scintillation Counter (Beckman Coulter, Fullerton, CA). Alternatively, the peaks were collected and subjected to a matrix-assisted laser desorption ionization time of flight (MALDI -TOF) MS. One microliter from each peak fraction was mixed with 9 μl matrix solution (a saturated solution of α-cyano-4-hydroxycinnamic acid and 0.1% TFA in 50% acetonitrile in water), and 1 μl of this mixture was spotted on a stainless steel plate. Mass spectra were acquired in positive ion mode with the reflector engaged on Bruker Daltonix Ultraflex III TOF/TOF mass spectrometer (Bruker, Billerica, MA). The Nd:YAG laser (355 nm) intensity was adjusted for optimum sensitivity and resolution. Single stage MALDI-TOF mass spectra were externally calibrated with a mixture of 7 peptides supplied by Bruker, ranging in monoisotopic *m/z *from 1046.54 (Angiotensin II) to 3147.47 (Somatostatin). The acceleration voltage was as follows: ion source 1 - 25.00 kV, ion source 2 - 21.90 kV, lens - 9.50 kV, reflector 1 - 26.30 kV, reflector 2 - 13.80 kV. Glycosylated hCG-β peptides' sequencing (TOF/TOF MS spectra) provided clear spectra with readily available -b/-y-ions that were analyzed with the Brukers' BioTools version 3.0 software.

### Purification of LTBMUC1 fusion protein

LTBMUC1 fusion protein was extracted from tobacco leaves by homogenization with cold 50 mM Tris HCl buffer pH 7.4 containing 0.1 M NaCl, 1 mM MgCl_2_, 3 mM DTT, and 1 mM PMSF. The homogenate was run through cheesecloth and centrifuged at 10 000 × g for 15 minutes at 4°C. The supernatant was saturated with (NH_4_)_2_SO_4 _to a final concentration of 75% (w/v) and incubated overnight at 4°C. The precipitated proteins were collected by centrifugation at 15 000 g for 45 min and 4°C, and the pellet was dissolved in a small amount of 50 mM Tris HCl buffer pH 7.4 containing 1 mM MgCl_2. _The samples were desalted using PD10 columns (Amersham Biosciences, Piscataway, MA), supplemented with NaCl to a final concentration of 0.1 M, and subjected to chromatography on Ni^2+ ^Fast Flow Sepharose (Amersham Biosciences, Piscataway, MA) according to manufacturer's recommendations. The eluted fractions were screened for the presence of the recombinant LTBMUC1 protein by SuperSignal West HisProbe Kit (Pierce, Rockford, IL). Blots were stained in parallel with Ponceau S (Sigma, St. Louis, MO).

### Lectin Analysis

Soluble proteins from tobacco leaves of the wild type or transgenic plants were extracted and (NH_4_)_2_SO_4_-precipitated as described above. After desalting using desalting spin columns (Pierce, Rockford, IL), 150 μl samples of total soluble proteins or purified LTBMUC1 normalized by Bradford assay [52] and Western blot/Densospot reading, respectively, were mixed in Zeba spin columns (Pierce, Rockford, IL) with equal volume of *Vicia villosa *(VVA) immobilized lectin gel (EY Laboratories, San Mateo, CA) pre-equilibrated with 50 mM Tris HCl buffer pH 7.4 containing 0.1 M NaCl and 1 mM MgCl_2_. The suspension was incubated overnight at 4°C on a Thermoline Labquake shaker. The non-bound proteins were then collected and the column was washed with 20 column volumes of buffer. Bound proteins were eluted with 50 mM Tris HCl buffer pH 7.4 containing 0.1 M NaCl, 1 mM MgCl_2 _and 0.3 M GalNAc, and used for further analyses.

SDS samples of VVA agarose-bound proteins were prepared, and SDS PAGE on 4-20% gradient polyacrylamide gels was performed as described earlier. The gels were either silver-stained using Silver Stain Kit (Pierce, Rockford, IL), probed with alkaline phosphatase-conjugated VVA (EY Laboratories, San Mateo, CA) following a protocol of Roche Molecular Biochemicals, or subjected to His-tag-based detection using SuperSignal West HisProbe kit (Pirece, Rockford, IL).

### Tandem mass spectrometry coupled to liquid chromatography (LC-MS/MS)

Following SDS-PAGE and silver staining as described above, the bands of interest were excised and digested with chymotrypsin (10 μg/ml) at 37°C overnight. LC-MS/MS analyses of in-gel chymotrypsin digested protein bands [53] were carried out using a linear quadrupole ion trap ThermoFinnigan LTQ mass spectrometer (San Jose, CA) equipped with a Michrom Paradigm MS4 HPLC, a SpectraSystems AS3000 autosampler, and a nanoelectrospray source. Peptides were eluted from a 15 cm pulled tip capillary column (100 um I.D. × 360 um O.D; 3-5 um tip opening) packed with 7 cm Vydac C18 (Hesperia, CA) material (5 μ, 300Å pore size), using a gradient of 0-65% solvent B (98% methanol/2% water/0.5% formic acid/0.01% triflouroacetic acid) over a 60-min period at a flow rate of 350 nl/min. The LTQ electrospray positive mode spray voltage was set at 1.6 kV, and the capillary temperature - at 180°C. Dependent data scanning was performed by the Xcalibur v 1.4 software [54] with a default charge of 2, an isolation width of 1.5 amu, an activation amplitude of 35%, an activation time of 30 msec, and a minimal signal of 100 ion counts. Global dependent data settings were as follows: reject mass width of 1.5 amu, dynamic exclusion enabled, exclusion mass width of 1.5 amu, repeat count of 1, repeat duration of 1 min, and exclusion duration of 5 min. Scan event series included one full scan with mass range of 350 - 2000 Da, followed by 3 dependent MS/MS scans of the nine most intense ions. Dynamic exclusion was set on with a time window of 5 min. Tandem MS spectra of the peptides were analyzed with TurboSEQUEST™, a program that allows correlation of the experimental tandem MS data with the theoretical spectra generated from known protein sequences [55]. The peak list (data files) for the search was generated by Bioworks 3.1 SR1. Parent peptide mass error tolerance was set at 1.5 amu and fragment ion mass tolerance was set at 0.5 amu during the search. Possible modifications such as alkylation of cysteine residues and oxidation of methionine residues as well as other possible modifications (e.g. +203 Da on threonine and serine residues), were included in the search parameters. The criteria that were used for a preliminary positive peptide identification were the same as previously described, namely peptide precursor ions with a +1 charge having a Xcorr >1.8, +2 - Xcorr > 2.5, and +3 - Xcorr > 3.5. A dCn score > 0.08 and a fragment ion ratio of experimental/theoretical >50% were also used as filtering criteria for reliable matched peptide identification [56,57]. All matched peptides were confirmed by visual examination of the spectra. All spectra were searched for initial protein identification against the non-redundant protein database downloaded from NCBI. The results were also validated with X!Tandem v. 2007.01.01.1 http://www.thegpm.org[58] and with Scaffold v. 2.0.3 (Proteome Software Inc., Portland, OR), a program that relies on various search engine results (ie: Sequest, X!Tandem, MASCOT), and which uses Bayesian statistics to reliably identify more spectra [59,60]. Peptide and protein identifications were accepted if the probability was greater than 90.0% as specified by the Peptide Prophet Algorithm [58] and the Protein Prophet Algorithm [60], respectively.

### Confocal microscopy

Leaves from tobacco plants expressing GalNAc-T2:GFP fusion protein were cut in half and incubated for 1 h at room temperature either in 50 mM Pipes buffer, pH 6.9 containing 5 mM EGTA and 5 mM MgSO_4_, or in the same buffer supplemented with 10 μg/ml Brefeldin A (Sigma, St. Louis, MO). Microscope slides were prepared with small segments of transformed leaves cut with a razor blade and mounted in water. Imaging was conducted on the Leica SP2 Multi-photon Scanning Laser Microscope (Leica Microsystems Inc., Bannockburn, IL) housed in the W.M. Keck Bioimaging Facility at Arizona State University. Multiple lasers allowed for simultaneous imaging of GFP fusion protein (Argon, 488 nm), and auto-fluorescence given off by the chloroplasts (Krypton, 568 nm). The detection channels (Ch) were set to the following ranges: Ch 1: 490-510 nm and Ch 2: 600-720 nm. Settings for gain and offset of the detectors were kept constant for all experiments. Using a 40× oil objective, images were scanned at 0.6 μm intervals in the z-axis. The post-acquisition image processing was performed using the Leica SP2 software (Leica Microsystems Inc., Bannockburn, IL) and Adobe Photoshop CS3 software (Mountain View, CA).

### In silico analyses

A GenBank/NCBI database http://www.ncbi.nlm.nih.gov was accessed in February, 2008 - May, 2009. Localization of proteins was predicted with PSORT software http://psort.ims.u-tokyo.ac.jp/form.html. Predictions of signal sequences and potential mucin type O-glycosylation sites were done with SignalP 3.0 http://www.cbs.dtu.dk/services/SignalP/ and NetOGlyc 3.1 http://www.cbs.dtu.dk/services/NetOGlyc/, respectively. A homology model of the tobacco endochitinase (GenBank Accession No AAA34070) was built based on the 3 D structure of *O. sativa *L. japonica class I chitinase (pdb ID 2DKV) using Geno3 D automatic molecular modeling tools http://pbil.ibcp.fr/htm/index.php and Jmol software http://molvis.sdsc.edu/fgij/index.htm for visualization.

## Competing interests

The authors declare that they have no competing interests.

## Authors' contributions

SD carried out the construction of pBYR:GNE.GT expression vector, transient plant transformations, radioactive enzyme assays, confocal microscopy, *in silico *analyses, LTBMUC1 purification and the subsequent molecular analyses, and drafted the manuscript; JR constructed pK7FWG2:GNT2 expression vector, carried out the non-radioactive enzyme assays, the molecular analyses of the transgenic GalNAc-T2 plants, and helped to draft the manuscript; ZC carried out the characterization of the reaction products of the enzymatic reactions by MS and peptide sequencing; SO participated in lectin chromatography and lectin blot analysis, prepared the endochitinase sample, and maintained the transgenic plants; GT performed nano LC/MS-MS analyses; HM constructed pBYsE1bE2T210 and pBYR-H2gpKDEL-K3 vectors, and provided magnICON 5' and integrase modules; LL constructed pH7WG2:GNT2 plant expression vector, generated stably transformed GalNAC-T2 plants, participated in molecular analyses, and conceived the study. All authors read and approved the final manuscript.
